# OA01.02. Associations between complementary and alternative medicine and conventional medical care utilization, access and quality of care

**DOI:** 10.1186/1472-6882-12-S1-O2

**Published:** 2012-06-12

**Authors:** C Bethell, N Gombojav, S Stumbo

**Affiliations:** 1 The Child and Adolescent Health Measurement Initiative (CAHMI), OHSU, Portland, USA

## Purpose

To assess complementary and alternative medicine (CAM) use in relation to utilization, access, parent’s experience with conventional medical care (CM) and CM expenditures for children with and without chronic conditions.

## Methods

Data sources used were the 2007 National Health Interview Survey (NHIS, n=9,417), the linked file of the 2007 NHIS to the 2008 Medical Expenditure Panel Survey (MEPS) (n=2,411) and the 2009/10 National Survey of Children with Special Health Care Needs (NS-CSHCN; n=40,243). Multivariate analyses were used to calculate adjusted odds ratios (AOR), controlling for children’s chronic condition and special needs status and demographic characteristics.

## Results

Nearly 40% of the over 1 in 5 children with special health care needs used CAM and nearly 45% of CSHCN using any CAM used two or more types. (AOR 2.54). Higher use of conventional medical care (CM) was associated with CAM use (AOR 1.94; NHIS/MEPS). Using both the NHIS and NS-CSHCN data, CAM use was more common among children who had difficulties accessing CM (AOR 1.73; NHIS). Among children with at least one chronic condition, parents who are less satisfied with doctor’s communication were more likely to use CAM for their children (AOR 2.20; NHIS/MEPS). The mean medical expenditures for CAM users were twice those of non-CAM users ($3,286 vs. $1,633, NHIS/MEPS). Variations in strength of associations were found by type of health condition and type of CAM used.

## Conclusion

CAM use is associated with the complexity and intensity of children’s health conditions and service needs, difficulties accessing CM services and experience with CM providers. Despite observation of important trends, condition specific analysis is limited by available data. Children with health conditions receive multiple forms of conventional, complementary and alternative care, emphasizing the need for well integrated and coordinated pediatric care systems.

